# Haecceity of Becoming-Mother: A Diffractive Analysis of Babywearing Through a Global Pandemic

**DOI:** 10.1177/15327086231169060

**Published:** 2023-05-02

**Authors:** Louise Platt

**Affiliations:** 1 Manchester Metropolitan University, UK

**Keywords:** babywearing, diffraction, haecceity, motherhood, pandemic

## Abstract

This paper problematizes notions of good/bad mothering by putting to work Deleuze and Guattari’s haecceity. Focusing on babywearing, it presents an autoethnography of my experiences of becoming-mother during the Covid-19 pandemic. Using diffractive analysis, I analyze the entanglement of my experiences of babywearing, research diaries, images/selfies, the regulations introduced during the pandemic, and the “global cultural script” of being a good mother. It works toward a position of (k)not knowing, being open to the unknown. Under the lens of haecceity, becoming-mother is “thisness”—a middle, knotting, and unknotting, ever emergent.

## Introduction

This paper addresses the haecceity (the *thisness*) of becoming-mother against the backdrop of the Covid-19 pandemic from the position of (k)not knowing (Osgood, 2019). This (k)not knowing has literal and metaphorical resonance. Focusing on babywearing as parenting practice, it presents an autoethnography of my experiences which sees a literal knotting of woven fabric around mine and my child’s bodies and metaphorically, recalling Osgood’s (2019) explanation of (k)not knowing, being open to the unknown and surprise. This openness is activated by employing a diffractive analysis where insights are read “through one another” (Barad, 2014, p. 25), specifically reading experiences of mothering during the pandemic through Deleuze and Guattari’s (2004) figuration of haecceity. I demonstrate how “mother” as becoming-mother—a middle, a folding and unfolding, knotting, and unknotting, ever emergent can be highlighted through the “radical specificity” of autoethnography (Sotirin, 2010) and the alternative philosophy of the subject that the lens of haecceity affords.

Indeed, becoming a mother has been established as an iterative emergence—a relational experience between our material and discursive engagements with the world where discourses of being a good or bad mother persist (Boyer & Spinney, 2016). As Faulkner (2014) so powerfully evokes, being a mother is complex and contradictory—therefore what does “good/bad” mean? In this paper, it is revealed that the lack of both formal (in-person visits from health visitors, breastfeeding support teams, or baby clinics) and informal (restrictions of family and friends visiting in person) support during the pandemic further challenged the attainment of being a so-called good mother (Güney-Frahm, 2020; Whiley et al., 2021). Corollary to this, with a focus on the materialities of babywearing, the paper builds on the work of Boyer (2018) and the role that matter plays in this becoming-mother. Furthermore, it brings the agentic force of media and political discourses during the Covid-19 pandemic in to the babywearing assemblage.

The paper seeks to pause events on the move and witness, “an intensive and fluctuating composite of force relations” (Sauvagnargues, 2013, p. 44) to resist the formation of a subject and seek an affirmative perspective of mothering in a pandemic. DeleuzoGuattarian philosophy, instantiated here using the concept of haecceity, demands that we conceive the world differently to live differently. The imperative of this way of thinking has been brought to the fore during the unprecedented times of living with Covid-19 (Harris & Holman Jones, 2021) through the radical shifts in our everyday lived experiences and through individual and collective trauma. Arundhati Roy’s (2020) notion of the pandemic as a portal is of relevance here, it offers a line of flight, a threshold (to use Deleuzian terminology) to understand everyday experiences of mothering anew. The intimate autoethnographic data utilized is important in understanding these experiences, for as Harris and Holman Jones (2021) comment, autoethnography is a space where the massive and the micro collide.

I employ haecceity as a tool to capture these forces and collisions in motion. Haecceity, for Deleuze and Guattari (2004), means *thisness*—it is unique in that moment—as such it is not anticipated or cannot be prepared for. It is neither object nor subject as a formed concept but an individuation—a becoming,

It is the entire assemblage in its individuated aggregate that is haecceity; it is this assemblage that is defined by longitude and a latitude, by speed and affects, independently of forms and subjects, which belong to another plane. (Deleuze & Guattari, 2004, p. 289)

A haecceity does not belong to a specific time-space but is dynamic. This paper analyses images and autoethnographic diaries that were recorded in chronological time (Chronos) but recasts them along floating time (Aion) where the two modes of temporality reveal a composition of becoming-mother that offers potentialities—“passages, propagations, and expansion” (Sotirin, 2005, p. 99). It leaves space for (k)not knowing.

## The Good Mother

Sociological and cultural conceptualizations of a good mother are most often associated with ideas of intensive mothering, a term coined by Hays (1996). As Kerrane et al. (2021) explore, intensive mothering centers the child, whereby the mother makes economic and social sacrifices. Indeed, even though intensive mothering has been identified as a hegemonic ideology (White, heterosexual, and middle class), these are not idealized ideologies (Johnston & Swanson, 2006) but characterized as “a global cultural script” for “proper” parenting (Kerrane et al., 2021, p. 1154). While it has been found that there is a difference between intensive behaviors and intensive attitudes (with attitudes not necessarily translating into behaviors) (Lankes, 2022), the implications of intensive mothering as a neoliberal project are far-reaching beyond one demographic with potentially damaging impacts on mothers mental health (Henderson et al., 2016). Lupton (2000) identifies a tension in the idea of good mother where romanticized notions of mothering eventually give way to the realization that, “mothering consisted of much repetitive, frustrating, arduous labour that often-lacked reward or recognition and from which it was difficult to escape because of the *good mother* ideal” (p. 58). This ambivalence is demonstrative of the binaries of good/bad mother and conservative versions of femininity and motherhood that permeate society (Budds et al., 2017). O’Reilly (2004) characterizes this ideology of mothering as oppressive and suggests a counter narrative of empowerment where mothering becomes a political site. Empowered mothering allows for autonomy and independence where radical challenge and change is possible. Despite this compelling alternative narrative, the dominant social norms of mothering persist such is the power of the patriarchal ideology (Johnston & Swanson, 2006; Smyth & Craig, 2017) and the neoliberal project that is intensive mothering. The discourses of good/bad mothering regulate women in how they present themselves, are seen by others, and feel about themselves. In developmental psychology, Burman (2016) traces the way in which these discourses come into being and ideal (and non-ideal) forms of mothering are “burdened by the weight of fantasy” (p. 152).

Furthermore, as McRobbie (2013) points out, neoliberal feminism has led to an alternative, but no less problematic, ideal vision of motherhood. The “housewife” becomes the “corporate family” with attendant aspirational lifestyles. New norms of middle-class life where “intensive investment in marriage, motherhood and domestic life” (McRobbie, 2013, p. 130) becomes a new form of feminine success. Whether mediated, as McRobbie (2013) explores, or evident in personal narrative (May, 2008), negotiating these social norms of mothering is something mothers inevitable internalize through self-critique and externalize through presentation. Harman and Cappellini (2015) identify that the presentation of mothering is often central where the *doing* rather than the *being* dominates. This draws on the influential work of Finch (2007) and family life as display. Finch (2007) suggests that display is central to how we *do* family and “fundamental to successfully constituting ‘my family relationships’ as a meaningful feature of my social world” (p. 79). Everyday family life is increasingly scrutinized in public domains leading to moralizing, where families are at once positioned as the problem and solution (Tarrant & Hall, 2020). Henderson et al. (2010) identify the constant pressure to be perfect is intensified under public scrutiny through modes of display which potentially contributes to “mother-blame.” Yet, they continue, this pressure emerges more strongly from interactions with other mothers and through forms of self-surveillance, “policing others and themselves” (Henderson et al., 2010, p. 241) rather than solely societal.

This public and private tension plays out on social media. “Sharenting” is grounded in pressures of good mothering (Lazard, 2022; Lazard et al., 2019). Referring to the development psychology outlined by Burman (2016), this work highlights narratives where the success of the child is perceived to be based on the success of the mother. Through posting family photos online, Lazard (2022) suggests that mothers are enacting digital identity repair work—to counter troubled identities offline. Yet, what is posted online is carefully managed to prevent accusations of “humblebragging.” Such is the power of the neoliberal discourse of good mothering, posting images online is identified as a “precarious, fragile and dynamic practice” (Lazard, 2022, p. 15) for women. She must at once display her neoliberal intensive mothering prowess while maintaining a non-competitive feminine image. Social media not only creates an opportunity for display but can be a place of support. However, Quinlan and Johnson (2020) illustrate the tensions of the public/private, expert/non expert that play out in these spaces using autoethnographic accounts of their own turn to social media in times of postpartum crisis. The online space provides access to information, and it also increases the pressure to perform a socially acceptable version of motherhood.

## Babywearing as a Parenting Practice

Babywearing, the practice of carrying your child in a sling or wrap, feeds into narrative of good mothering. Indeed, the practice is often associated with “attachment parenting” a philosophy parenting that promotes physical closeness between parent and child to create a responsive style of parenting (Granju & Kennedy, 1999) which also includes breastfeeding and co-sleeping. The benefits of babywearing are aligned to the importance of touch and the scientific evidence of “skin to skin care” (Reynolds Miller, 2016; Reynolds Miller et al., 2020). The role of the bodies of the mother and child are centralized here and Hallenbeck (2018) points out that while babywearing disrupts the usual mother-pram assemblage and offers the potential for “feminist world-making” (p. 367), this aspect remains underexplored.

Williams and Turner (2020) identify carriers are often only marketed as “travel devices” with their bonding capacity potentially diminished in the marketplace. However, the skill and investment required to practice babywearing could fall under the “intensive” descriptor. There is a proliferation of babywearing advice groups on social media leading to anxieties of “doing it right” or using the correct sling/wrap. The practice thus has implications for how mothering is conceived of and experienced:

Though the activity cultivates new assemblages in time and space by situating mothers and babies anew within the world and enabling greater maternal mobility, it also makes new demands of them. (Hallenbeck, 2018, p. 366)

Arguably, as a less obvious form of extensive mothering (Christopher, 2012), Russell (2015, p. 136) identifies that in the west, in the absence of a cultural tradition of babywearing, education is needed and has manifest in an “outsourcing of authority” from mothers to “babywearing consultants” (an official title gained on completion of accredited training from The School of Babywearing). In her study of Black mothers and attachment parenting, Hamilton (2020) identifies that babywearing as a cultural practice has been conflated with western ideas of natural parenting (p. 100). She states that “the resurgence of interest in babywearing operates as both a scientization of what should be a natural activity and an appropriation of African traditions.” In the context of this paper, the Covid crisis led to the closure of all support services and therefore the education identified by Russell (2015) and Whittle (2019) was not available in person which meant new parents could not access support from experts and thus had to seek alternative sources of information (usually online) that may not be promoting the safest tools and techniques, or leave them exposed to criticism in online forums.

There are a range of carriers available from structured, buckles, half-buckles, ring slings, woven wrap, mai tai, onbuhimo, and all come with their own challenges, suiting different bodies or circumstances, and holding their own cultural meaning. Djohari (2016) reveals the agentic power of carriers as an object. She examines how the physical structure of the woven wrap—its malleability, the “give,” how it feels to work with, is enmeshed with feelings of sentimentality linked to its intimate usage—each wrap possessing its own character. This focus on the material of the carrier/wrap has thus far been under-examined, especially in relation to the intrinsic embodiment of carrying a baby. Indeed, the material culture of babies has generally received limited attention. Orrmalm (2020), focusing on the material of baby socks, highlights that babies’ interactions with material culture is important in understanding how they create relations with objects beyond meanings and functions. What this perspective offers is an understanding that a parent’s and child’s interaction with the same material culture, while maybe co-created through “inter-embodiment” (Lupton, 2013) is their own unique experiences.

## Haecceity and Becoming-Mother

Becoming-mother has been examined previously (Boyer, 2018; Boyer & Spinney, 2016; Clement & Waitt, 2018) but by putting haecceity to work I want to offer an alternate rendering which will contribute to an emergent idea of mother and mothering. St Pierre (2017) argues that one does not *apply* haecceity. Rather, I am putting it to work (Jackson & Mazzei, 2012) to reveal an alternate understanding of an endless emergence of motherhood rather than the formation of a static subject or body; “a perpetual disordering and perturbation of order, an incessant dislocation and scrambling of codes, and a ceaseless destabilisation on the move” (Doel, 1996, p. 427). In this regard, Deleuze and Guattari (2004) explain that the proper name (in this case *mother*) is merely a “diagram of an assemblage” (p. 292)—a becoming rather than a form taking. To take this perspective resists fixing identity and the formation of a subject—a haecceity is indeed more than the subject. St Pierre (1997) comments that “the subject no longer remains separate from objects or time or space but enters into composition with them” (p. 412). And in doing so, infinite possibilities emerge as haecceity. This is about intensities and “explodes the ideas about what we are and what we can be” (Sotirin, 2005, p. 99).

To do this, we need to turn attention to the DeleuzoGuattarian ideas of the molar and the molecular. The molar (e.g., the social intuitions such as ideas of good mother) is not replaced by the molecular (e.g., the singularities or lines of flight) but a potential re/de-territorialization. Merriman (2019) explains that becoming-molecular and becoming-molar is about movements and affects, where things shift between imperceptible and perceptible (p. 67). Molecular movements he states are “vital, incessant and unruly” but they cut across molar organizations and stability. They co-exist. Haecceity, Sauvagnargues (2013) explains, offers a moment in time to capture these molar and molecular forces as they move. As such, the analysis presented here is suggesting haecceity can offer a potential to deterritorialize the molar to develop new, emergent figurations of mothering. So, becoming-mother *might* be an escape from the social norms of good mothering, but it is also an individuation—becoming-mother is a haecceity. It is *this*. Of itself.

## Diffractive Analysis of Babywearing

In the 12 months since the start of the UK National lockdown in March 2020, Manchester, England, had been in some version of enhanced lockdown for over 300 days due to global COVID-19 pandemic. Amid this however, life quite literally went on, and I gave birth to a boy in the May 2020. What emerged, became something unexpected. I was on maternity leave and not consciously “researching” or “gathering” data apart from jotting a few notes down into the tiny screen of my phone at night concerning my thoughts about walking in my local area with my son. This was somewhat of an accidental autoethnography, yet it possesses a “radical specificity” (Sotirin, 2010). I did not set out to conduct an autoethnography of babywearing—I was, unwittingly, engaging with a (k)not knowing during my time caring for my new baby. In the same way, St Pierre (2017) came to realize that haecceity opened an alternative perspective on understanding identities, I likewise was “jarred” (Ringrose & Renold, 2019) by the “data” when seen through the lens of the haecceity. In the (post)qualitative turn, there is an acknowledgment that “data have their ways of making themselves intelligible to us” (MacLure, 2013, p. 660), and this paper emerged after periods of intense frustration of trying to tell this story—an embodiment of writing-as-inquiry (Richardson, 2003). It began life as a paper concerning walking, but on returning to Deleuze and Guattari’s (2004)*A Thousand Plateaus* to extend my thinking on becoming, I was “jarred” by the figuration of haecceity and what potential it offered.

Therefore, what surfaces is a diffractive analysis that seeks to account for the entanglements of engaging in and with the world. Like Haecceity, a diffractive analysis helps us to “think otherwise” (paraphrasing Lather, 2016, p. 126). Under her onto-epistemological framework, where knowing and being are entangled, Barad (2007) explains that diffraction “provides a way of attending to the entanglements in reading important insights and approaches through one another” (p. 30). Specifically, Mazzei (2014) explains that a diffractive analysis moves away from a normative, humanistic method of coding, interpreting, and representing. Using diffraction as methodology responds to the perspective of the subject as emergent through ongoing intra-actions where the material and the discursive are mutually constituted. Barad (2007) comments that, “diffraction does not fix what is the object and what is the subject in advance” (p. 30). Haecceity as a generative figuration here is just one potential among many and this approach allows space for potentialities, “seeing how something different comes to matter” (Davies, 2014, p. 734). Indeed, as Davies (2014) further identified, “It involves hard epistemological, ontological and ethical work to enable the not-yet-known to emerge in the spaces of the research encounter” (p. 735). A diffractive analysis therefore avoids what Haraway (1988) refers to as the “god trick”—we are not separate to the world but are part of it and this becomes evident in the endless knotting and unknotting of the autoethnographic accounts that are utilized.

To enact this diffractive analysis, I drew on a range of affective and material intra-actions. The images used below were taken on my phone—usually as a “selfie” due to the fact I was at home or out on a walk/errand with only my new baby. Some of these images are posed, ready to post on Instagram or via Whats App messages to share my experiences with friends and family who could not be near us—a performance of motherhood for an audience (Finch, 2007). Some pictures are more functional in that I would take a photo to examine the position of my son in the carrier and whether I had wrapped him correctly—still a form of display but where the emphasis is less on that *Kodak moment*. None of these images were “data” until I embarked on the writing. When I began to think about my babywearing practice, an emotionally affective practice, I decided to shift the images from the context they were taken and disorientate my thinking about them through an encounter with haecceity. I found printing them out and looking at them laid out across my desk gave me a different perspective to scrolling chronologically on my phone—moving them around as an iterative process of making and remaking their meanings. Furthermore, my babywearing practice was not simply assigned to images—I am still knotting and unknotting my child as he grows. Therefore, my constant engagement with babywearing while writing is folded into this emergent analysis.

I had made notes, mainly during the night into my phone while sitting in bed next to a sleeping baby. These autoethnographic diaries were not structured, but were memories and feelings jotted down in a moment of peace. Indeed, at the time they were not destined for an academic paper but as Harris and Holman Jones (2021) state, the pandemic has altered how we all see ourselves and the world we inhabit,

And perhaps this now-ness, this this-ness, isn’t just for autoethnographers because we are all—each of us—reckoning with our infinitesimal selves, and relation to the infinite pandemic. (p. 862)

The affective nature of the pandemic and subsequent lockdowns was also, “an energy that moves things” (Harris & Holman Jones, 2021, p. 868). These things, my “data,” cannot be disentangled from the *vibrancy* of COVID-19 (Sikka, 2021). Media and political discourse permeated into our everyday lives during this time. Especially before my son was born, in those last few weeks, I spent hours watching the news. Later, trying to unravel the “rules” and what they meant for me and my family. As such, often, all ethnographers “have available are their memories, and therefore, recalled events, conversations, feelings, and experiences constitute the material on which the autoethnography is built” (Winkler, 2018, p. 238). Therefore, it is not linear like a traditional research diary might be. In particular, the embodied, non-representational, nature of the “data” cannot be written down—it becomes “sticky data . . . that hovers, gnaws, prods and teases us” (MacRae et al., 2018, p. 504). The feelings that linger in the postpartum body, the sensations of carrying a child in a wrap, the energy exerted to wrap and knot efficiently—these are bodily, and while description can be attempted this “data” is sensuous—haptic knowledges (Paterson, 2009) being produced in the moment of doing.

This diffractive analysis thus breaks down binaries and can “trouble dichotomies” (Barad, 2014, p. 168) so that good/bad mother might be undone and reimagined. In constructing the below, I enacted agential cuts (Barad, 2014, p. 2007) to reveal haecceities of becoming-mother and like Warfield (2019, p. 154) “. . . I am choosing moments when my adherence to those values was shaken, disrupted, unsettled, and challenged”—where the values are my own desire to be a “good mother.” This “cutting together-apart” (Barad, 2014) becomes an entanglement of moments and matter, theory, and autoethnographic vignettes. As explained previously, these cuts are just potentials and passages—among many potentials and passages that might have been. An agential cut is merely a momentary stabilization.

## Haecceities of Becoming-Mother

In what follows I identify, emerging from the diffractive analysis, narratives of molecular intensities which contribute to the emergent haecceities of becoming-mother. The haecceities provide micro insights into the life-changing experience of becoming-mother. The use of images and a diffractive reading of them offer a *line of flight* into the “perpetual disordering” (Doel, 1996, p. 427), a (k)not knowing of how to be a good mother during a pandemic. The context for this narrative derives from the message that came from the UK Government in March 2020 to stay at home. When our son arrived in early May, we stayed at home, just us—myself, my partner, our newborn son, and our elderly dog. Family unable to cross the threshold, or even hold our son. So, I held him close.

### Baby-Mother-Wrap-Rules: Assemblages of Mothering

**Figure 1.**
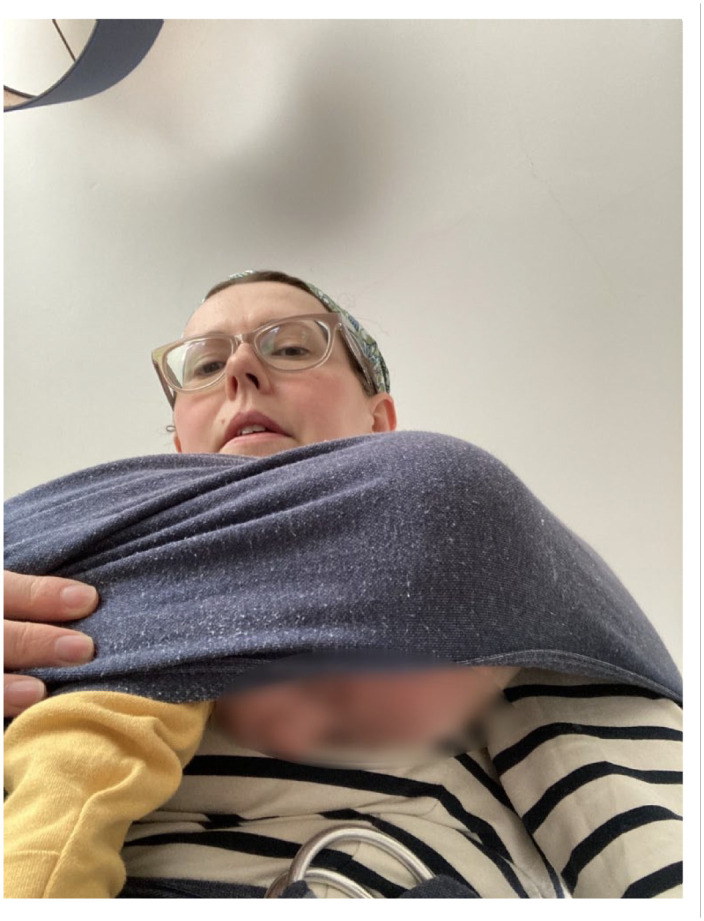
We Have Time to Get it Right . . . next slide please

The process of babywearing, particularly using a woven wrap, reveals the unruly bodies of a baby and a post-partum woman. Babywearing is an enmeshing of bodies. This (inter)embodiment (Lupton, 2013, p. 39) is an entanglement between the caregiver and the baby where autonomous bodies are literally intertwined and “lived alongside and in response to” each other (Figure 1).

The bodies of the child and the mother are pressed together but neither body is static, each move and writhes—resisting each other and the fabric:

He is a leaner and a leg straightener. I stumble along the uneven path holding him under his bottom to stop him falling the ground. When I tied off this carry I messed up—maybe today was not the day to experiment with a new carry. He pushes his legs downwards and throws his head back. “Come on kiddo, just stay still until I can get you sorted.” We arrive at the chemist, sweating (me, not him). Masked up and trying to readjust the wrap whilst staying distanced from others waiting in line.

This . . .

this . . .

this . . . was-not-the-day.

I just want to get my flu jab and get home. She asks me to stay in the area for 10 mins in case of side effects. I am hot, my glasses have steamed up and he is not impressed. He wants his arms freeing. He starts to twist against my body. We must leave now.

Utilizing babywearing in attachment parenting is not straightforward as the above demonstrates, but the relations between bodies is revealing. The child has its own relations with the material and the body of the mother. They are not passive passengers, along for the ride. As Lupton (2013) explores, the infant has the capacity to disorder—his body twists and resists the wrap and my pleas to stay still. He has no desire to be held close in this moment. The idealized vision of the perfect model of attachment parent is not evident in this narrative above—the mother here is sweaty, she has lost control of the child, she is impatient. Yet it is in this “perpetual disordering” (Doel, 1996, p. 427) that relations are formed, and understandings are reached. We are learning to co-exist and the intensities belong to us—mine, his, and mine-and-his, they are lives happening and bonds forming in motion. Deleuze and Guattari ask not what the body means but what the body can do. We need to rephrase that here as “what can the *bodies* do?” We are not seeking to understand what the bodies mean in this haecceity—the mother body, the infant boy body—but what the entire assemblage might produce in that moment.

This assemblage takes in the media discourses during the pandemic and the rules and regulations by which we were all asked to live by. These narratives were inscribed on bodies and became part of the assemblage and thus my becoming-mother during this time:

It should be read without a pause. (Deleuze & Guattari, 2004, p. 290)

The-mother-carries-the-baby-in-the-rain

A-baby-counts-as-a-person-in-the-rule-of-six

Children-under-school-age-do-not-count-in-two-person-limit

The assemblage of mothering encountering the assemblages of a pandemic, playing out in media discourses, was my early mothering experience. Spending large parts of my evening sat in bed next to a sleeping child, scouring the regulations just announced. Trying to grasp what was and was not allowed:

What a life! What a lockdown! (a very singular individuation) . . . to paraphrase Deleuze and Guattari. (2004, p. 288)

**Figure 2.**
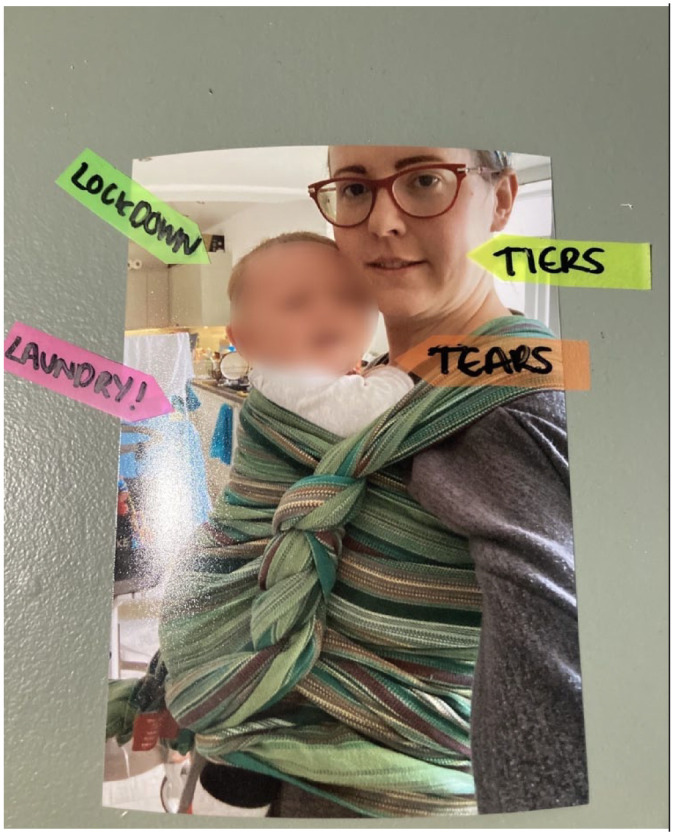
A Fancy Finish?

Societal pressures to be a good mother pervades the mundane but also seeps into the unprecedented times. Wearing my child around the house out of necessity to get jobs done (to enact McRobbie’s notion of corporate family life) is also an embodiment of the stay-at-home messaging that contained my mothering. Figure 2 demonstrates a seemingly paradoxical mothering. Knotting a “fancy finish” while (k)not knowing how and when the rules and regulations might change. In October 2020, the government announced varying regional restrictions in England known as tier regulations with alert levels of medium (tier 1), high (tier 2), and very high (tier 3). The announcement of Greater Manchester being place in in tier 3 produced tears, but the laundry still needed to be done. The knotting of the wrap was also a knotting in the stomach as panic set in of raising a child with no involvement from formal or informal support mechanisms. However, a diffractive analysis using haecceity allows a different perspective on the chronology of time and the evolving regulations along that timeline. Indeed, it is now hard to recollect which rules happened when—indeed, as I write this section, I find myself “Googling” when different regulations came into force. In this diffractive analysis, we can witness shifts between Chronos and Aion where Aion time “traces the frontier between things” (Dewsberry, 2000, p. 480) so that mothering has multiple pasts and futures—it is all that has been and could be, always a becoming. As time passed, new rules came and went, my mothering of course evolved in real time—from day to day, but never a fixing at each point in time as it is also in motion. My becoming-mother did not “happen” but was (is) “happening” as the pandemic rules influenced our daily lives.

Not only does a haecceity reconfigure our notions of time, there is an intersection of latitudes and longitudes, a “middle”—not a middle in the sense of a balancing point or compromise, but a middle in terms of in process. To attune to the latitude of the haecceity, we see the power to affect or be affected. These “jarred” me as key affective moments:

You are wrapped up secured in a beautiful woven wrap that was maybe a bit of a splurge but too gorgeous to resist. We already have carriers but this 4.6m of green cotton woven fabric will allow us to walk, body to body, for much longer. The chance to wander freely, wave at people from a distance, get my weary bones moving—brush off that cooped up feeling that creeps in (when the health visitor check on your mental health you try and tell her you’re ok.

But

you’re

not.

But

you

are.

It’s too complicated to even process yourself just yet). I unravel you. Carefully supporting you and lowering you into the cot—trying not to wake you. The walk has rocked you to sleep.

We

know

we

will

do

it

all

again

tomorrow.

And

for

the

days

to

come.

This . . . This . . . This . . .

**Figure 3.**
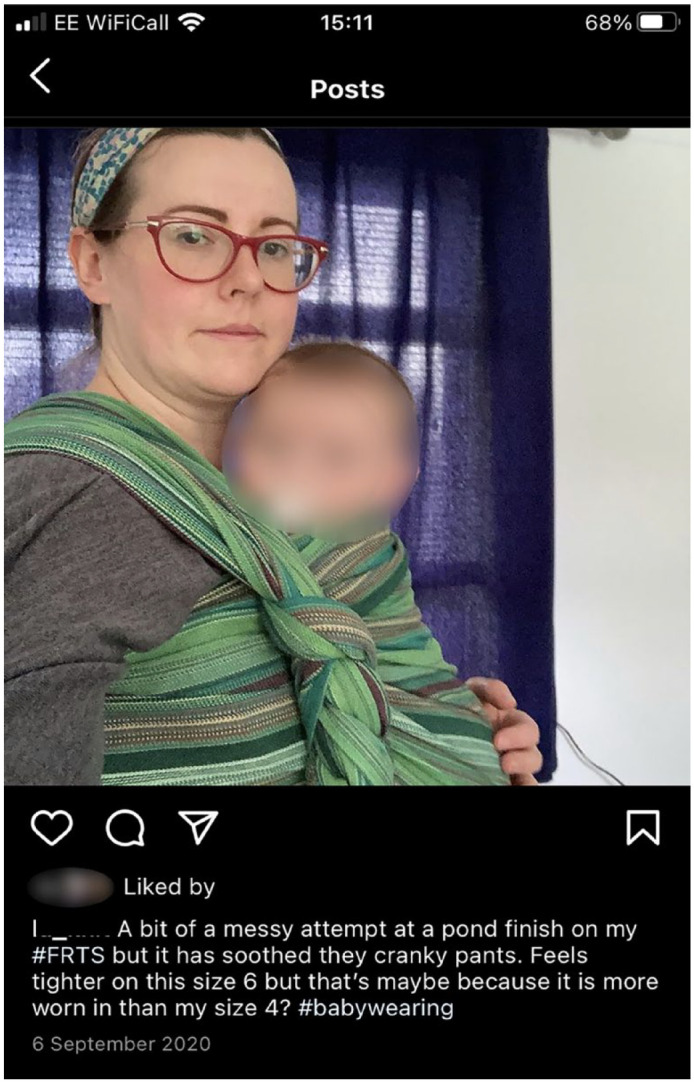
“It Is Not Made of Points, Only Lines. It Is a Rhizome” (Deleuze & Guattari, 2004, p. 290).

The individuation of mother that emerges here is not permanent, as established above, but it is a middle—like a stream that picks up speed, it “sweeps one *and* the other away” (Deleuze & Guattari, 2004, p. 28). It is never an end point where I emerge as a version of good (or even bad) mother. Babywearing, like my mothering, was a process. It still is, as I continue the practice of carrying even as my son grows into a toddler. Figure 3 depicts the “pond finish,” an elaborate twisting and knotting of the wrap to create both a secure fasten and a “fancy finish” that looked pleasing. In relational thinking, the body is constituted through relations with other things. My (k)not knowing of both how to be a mother and how to wrap safely is entwined.

### Displaying Mothering

I unravel you. Carefully supporting you and lowering you into the travel cot—trying not to wake you. The walk has rocked you to sleep. We know we will do it all again tomorrow. For as long as outside is the only place we can show you off.

During the pandemic, I was “left in the dark” with regard to what a good mother might look like. This led to heightened anxieties and confusion. This was channeled into babywearing and a determination to do it correctly, especially back carrying. I would obsessively watch YouTube videos to perfect my techniques. The need to keep the child safe is the virtue of a good mother slavishly enacted. Learning from videos to master “the hip scooch” method of getting my son onto my back (a method of maneuvering the child around from the front of your body, over your hip, and securing them onto your back in a safe way) was imitation—it was copying. But, as haecceity it was also its own thing—it was my body and his body and the endless knotting and unknotting of the fabric. Positioning and repositioning of both our bodies. The haecceities of trying to back carry are non-representational, they are not about imitating the perfect mother but a refrain of an emergent becoming-mother.

**Figure 4.**
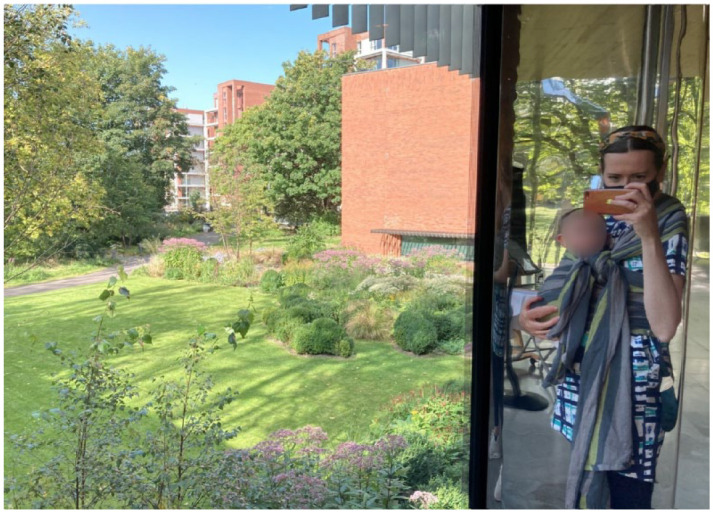
Practice Makes Perfect. YouTube . . . “How to Tie a Slip Knot.” . . . Museums Are Open. Finally, I Can Show Off My Front Reinforced Traditional Sling Carry #FRTS.

The search for the perfect wrap, to execute a back carry, in my mind was tied to my quest to be the “good mother” I had imagined I would be pre-covid. Being at home, with nowhere to go, this preoccupation filled the void. As explored, display is an important aspect of mothering and feeling validated as a good mother. During lockdown, opportunities for display were limited to family on video call or posting photos online. The pandemic had taken away the opportunity to share my new baby with the world. Initially, I was unable to even show him off to my family. If display is how we *do* family (Finch, 2007), this had to be reimagined under the circumstances of the pandemic. Taking my son to the museums and galleries I loved was at top of my maternity leave agenda and when they temporarily reopened in the late Summer of 2020, we made the journey into the city center. Abandoning the pram in the gallery foyer, I was excited to wrap him and share these spaces with him (Figure 4).

**Figure 5.**
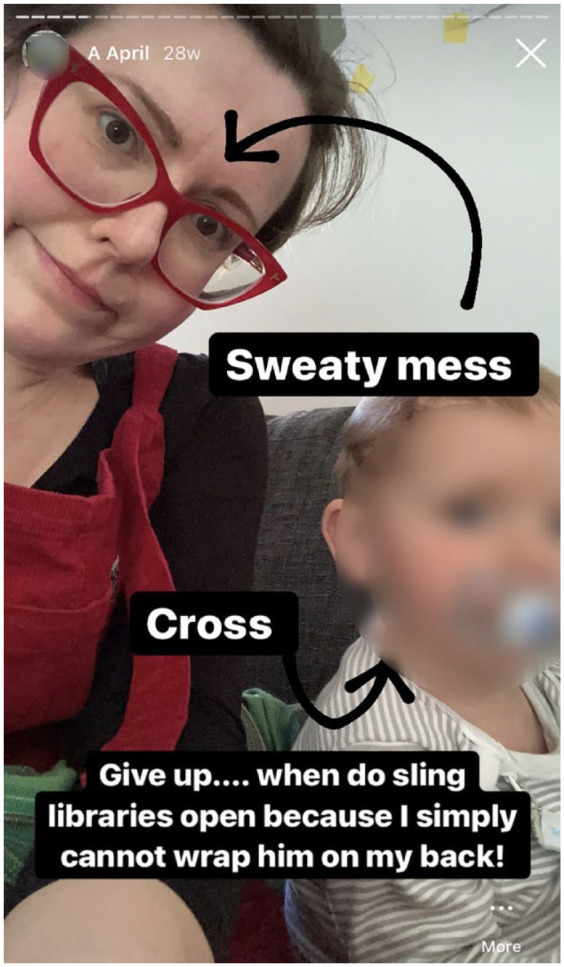
**#**Icried. #Ifailed.

I was excited to be seen in a public space, babywearing (not least as I had perfected a new carry). Being seen, witnessed, validated my mothering—my good mothering. However, as explored above, display in both online and offline spaces is complex for the mother for whom societal pressures demand certain performances. My images of babywearing demonstrate the tensions outlined by Lazard (2022). A balance of wanting to demonstrate my babywearing prowess while avoiding the “humblebrag.” Figure 5 is illustrative of that. What seems to be a light-hearted image of the aftermath of another attempt of back carrying jarred me on reviewing retrospectively. I recall crying out of frustration after posting this picture.

My failure to back carry is internalized in the moment as a failure to mother. However, this image, this display on social media, does not account for the refrain of babywearing. The repetition with difference, the constant, endless mothering in motion. My mothering is always a line of flight. It is the movement of the molecular away from the molar. It is captured in the haecceity.

**Figure 6.**
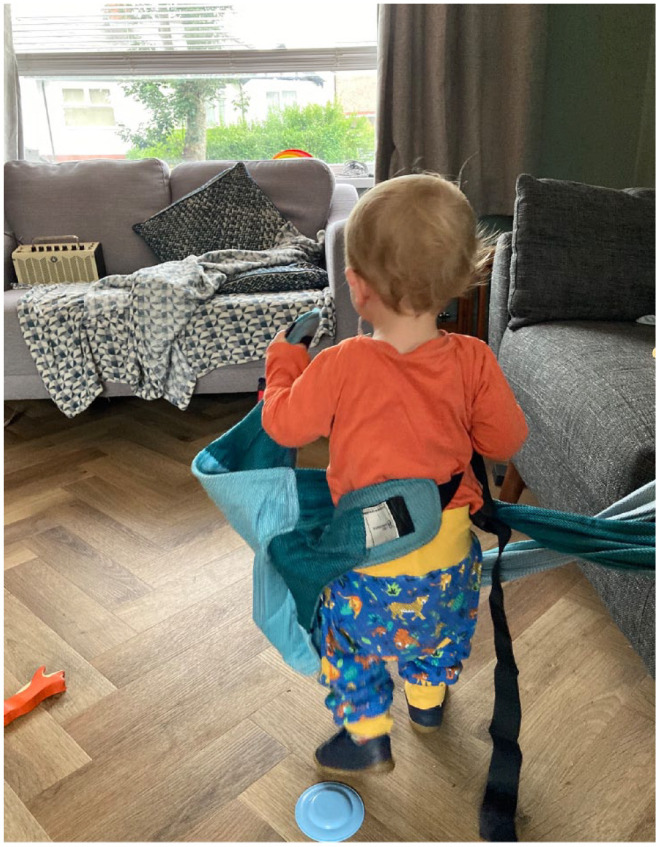
“Becoming Is Never Imitating” (Deleuze & Guattari, 2004: 336).

I have spoken of imitating what babywearing should look like, thus what I thought mothering should look like (Figure 6). MacLure and MacRae (2022) read the Deleuzian idea of imitating as a folding and mutually elaborating activity of the inner world of the subject and outer worlds of matter. Imitation is generative then, a becoming something new. So, through each display of mothering, trying to find validation in my being a good mother (by societal standards), I am becoming-mother not as a sameness but as a difference. Deleuze and Guattari (2004) talk of becoming-animal, and in imitating the “molar” dog (the fixed notion of dog) you emit the “molecular” dog. Therefore, my imitating the idea of good mother through display is emergent and generative of a unique becoming-mother. Mothering is de/re-territorialized through my imitating and display.

## Conclusion

What constitutes a good mother is problematized in this diffractive analysis. As Taylor (2016) suggests, this analysis pays “greater attention to research as an emergent enactment of materially embodied socio-political practices, and to the cuts, boundaries, and differences we co-constitutively produce through knowledge enactments” (p. 203). Therefore, the simple binary of good/bad is challenged when encountered through haecceity and a middle, an emergent subject is sought. Cummins and Brannon (2021) identified that the pressure of intensive mothering was further compounded during the pandemic. They conclude that if ideas of good mothering are societal and normative, then the experiences of mothering during a pandemic needs further attention to potentially rethink these definitions. The haecceities are expressions of becoming and embody changing states—in this case, endlessly transitioning to becoming-mother.

The individuation of the mother as a subject is prevalent in narratives of being a competitive participant in the workforce and a neoliberal emphasis on the individual but when examined as “thisness” the mother-child-wrap assemblage in this paper reveals an entanglement where these competing forces are neither good nor bad. The push and pull of becoming our own versions of something is more than the subject. Therefore, the call of Sotirin (2010) for a more radically specific autoethnography is met here in the pandemic portal (Roy, 2020) to present an affirmative (k)not knowing (Osgood, 2019). It offers an alternative which might untether us from fixed concepts and definitions and encourage us to capture life in motion.
